# Surgical Versus Dilational Tracheostomy in Patients with Severe Stroke: A SETPOINT2 Post hoc Analysis

**DOI:** 10.1007/s12028-023-01933-9

**Published:** 2024-01-30

**Authors:** Hauke Schneider, Jan Meis, Christina Klose, Peter Ratzka, Wolf-Dirk Niesen, David B. Seder, Julian Bösel, for the SETPOINT2 and IGNITE study groups

**Affiliations:** 1 Department of Neurology, University Hospital Augsburg, Stenglinstr. 2, 86156 Augsburg, Germany; 2 Medical Faculty, University of Dresden, Dresden, Germany; 3 Institute of Medical Biometry, University of Heidelberg, Heidelberg, Germany; 4 Department of Neurology and Neurophysiology, Medical Center - University of Freiburg, Faculty of Medicine, University of Freiburg, Freiburg, Germany; 5 Department of Critical Care Services, Maine Medical Center, Portland, ME USA; 6 University of Heidelberg, Heidelberg, Germany; 7 Johns Hopkins University Hospital, Baltimore, MD USA

**Keywords:** Stroke, Ischemic stroke, Intracerebral hemorrhage, Subarachnoid hemorrhage, Tracheostomy, Decannulation

## Abstract

**Background:**

Tracheostomy in mechanically ventilated patients with severe stroke can be performed surgically or dilationally. Prospective data comparing both methods in patients with stroke are scarce. The randomized Stroke-Related Early Tracheostomy vs Prolonged Orotracheal Intubation in Neurocritical Care Trial2 (SETPOINT2) assigned 382 mechanically ventilated patients with stroke to early tracheostomy versus extubation or standard tracheostomy. Surgical tracheostomy (ST) was performed in 41 of 307 SETPOINT2 patients, and the majority received dilational tracheostomy (DT). We aimed to compare ST and DT in these patients with patients.

**Methods:**

All SETPOINT2 patients with ST were compared with a control group of patients with stroke undergoing DT (1:2), selected by propensity score matching that included the factors stroke type, SETPOINT2 randomization group, Stroke Early Tracheostomy score, patient age, and premorbid functional status. Successful decannulation was the primary outcome, and secondary outcome parameters included functional outcome at 6 months and adverse events attributable to tracheostomy. Potential predictors of decannulation were evaluated by regression analysis.

**Results:**

Baseline characteristics were comparable in the two groups of patients with stroke undergoing ST (*n* = 41) and matched patients with stroke undergoing DT (*n* = 82). Tracheostomy was performed significantly later in the ST group than in the DT group (median 9 [interquartile range {IQR} 5–12] vs. 9 [IQR 4–11] days after intubation, *p* = 0.025). Patients with ST were mechanically ventilated longer (median 19 [IQR 17–24] vs.14 [IQR 11–19] days, *p* = 0.008) and stayed in the intensive care unit longer (median 23 [IQR 16–27] vs. 17 [IQR 13–24] days, *p* = 0.047), compared with patients with DT. The intrahospital infection rate was significantly higher in the ST group compared to the DT group (14.6% vs. 1.2%, *p* = 0.002). At 6 months, decannulation rates (56% vs. 61%), functional outcomes, and mortality were not different. However, decannulation was performed later in the ST group compared to the DT group (median 81 [IQR 66–149] vs. 58 [IQR 32–77] days, *p* = 0.004). Higher baseline Stroke Early Tracheostomy score negatively predicted decannulation.

**Conclusions:**

In ventilated patients with severe stroke in need of tracheostomy, surgical and dilational methods are associated with comparable decannulation rate and functional outcome at 6 months. However, ST was associated with longer time to decannulation and higher rates of early infections, supporting the dilational approach to tracheostomy in ventilated patients with stroke.

**Supplementary Information:**

The online version contains supplementary material available at 10.1007/s12028-023-01933-9.

## Introduction

Tracheostomy, that is, securing the airway tracheal cannula, is frequently necessary in patients with severe stroke requiring critical care and mechanical ventilation. Dilational tracheostomy (DT) can be performed at the bedside and with low rates of early complications if performed by experienced physicians [1, 2]. Surgical tracheostomy (ST), performed by surgical teams in the operating room or at the bedside, can provide patients with a more stable tracheal stoma, but it often necessitates a surgical closure after decannulation.

A Cochrane review in 2016 compared dilational with surgical techniques for tracheostomy, including several earlier randomized trials that mainly recruited patients without stroke [3–5]. The quality of evidence of the identified studies was mainly low, and procedures were performed by a wide range of differently experienced operators. There was no difference in primary outcomes between ST and DT groups, for intstance, the mortality rate directly related to the procedure and the rates of intraoperative or direct postoperative complications were comparable. However, patients undergoing DT had fewer wound infections and less unfavorable scarring. For other secondary outcomes (major bleeding, tracheostomy tube occlusion, accidental decannulation), no differences were found. Periprocedural tracheostomy complications were observed in 3–7% (e.g., bleeding, wound infections) of patients overall [6, 7].

Data on long-term outcomes and on participant-relevant outcomes after tracheostomy in patients with stroke are often sparsely reported or not available [3].This includes frequencies of decannulation, time to decannulation, and rates of long-term complications of tracheostomy, such as dysphagia, local pain, tracheomalacia, and impaired wound healing. In a prospective single-center study, 59% of tracheotomized patients with stroke were decannulated at 12 months. Decannulated patients had an improved functional outcome compared to surviving patients without cannula removal (median Barthel index 35 vs. 5 [*p* < 0.001]), suggesting that decannulation may serve as possible surrogate outcome parameter in patients with stroke [8]. In a current meta-analysis including 451 patients, 30% of patients achieved functional independence and 36% survived in a dependent state; decannulation occurred in 79% of surviving patients [9–12]. In a recent retrospective single-center study including neurosurgically treated patients, 40% of trachotomized patients were decannulated within 6 months (median 89 [interquartile range {IQR} 59–133] days).

The Stroke-Related Early Tracheostomy vs Prolonged Orotracheal Intubation in Neurocritical Care Trial 2 (SETPOINT2) trial randomly assigned 382 mechanically ventilated patients with severe stroke predicted to require tracheostomy to either an early approach to tracheostomy or to a standard approach, including extubation or tracheostomy, if needed [13, 14]. Six months after stroke onset, there were no differences of functional outcome between the groups. DT was the preferred method according to the trial protocol, however, ST was also permitted and was performed in a substantial number of trial participants. Within the subgroup of tracheostomized patients (*n* = 307), 41 patients received an ST.

We aimed to compare patients of the SETPOINT2 trial treated with ST and those treated with DT, matched for stroke type and patient age at stroke onset. The frequency of decannulation within the first 6 months after stroke onset was defined as the primary outcome.

## Methods

### Study Design

This was a post hoc analysis of the recently published SETPOINT2 randomized trial that included the blinded outcome evaluation of functional outcome at 6 months [13].

### Patients

The investigator-initiated, randomized controlled trial SETPOINT2 (ClinicalTrials.gov NCT02377167) recruited 382 ventilated patients with stroke in university centers in Germany and the United States. Participating trial centers were largely academic neurocritical care units and had to primarily employ DT techniques as their standard of care.

Patient selection criteria included severe stroke (acute ischemic stroke [AIS], intracerebral hemorrhage [ICH], or subarachnoid hemorrhage [SAH]), the need for prolonged ventilation and tracheostomy (as defined by both the opinion of the treating intensivists and a Stroke Early Trachestomy score [SETscore] [15] of > 10 points), and the ability to perform a tracheostomy within 5 days after intubation. Exclusion criteria comprised specified contraindications to a DT (e.g., anatomical distortion of the neck; previous surgery, burns, or radiotherapy to the neck; tracheal distortion, stenosis, or malacia; and morbid obesity) and the expected need for a permanent ST (e.g., for clinical reasons unrelated to the stroke or due to extensive destruction of the brainstem).

Trial participants were randomly assigned 1:1 to either the early group receiving tracheostomy as soon as feasible and within 5 days after intubation (“early tracheostomy”) or the control group (“standard of care” group), in which tracheostomy was performed ≥ 10 days from intubation if weaning and extubation was not feasible or successful. The primary outcome of the SETPOINT2 trial was the score of the modified Rankin Scale (mRS) at 6 months, and an acceptable outcome was defined as an mRS score of 0–4; investigators scoring the mRS were anonymized to tracheostomy type (intervention). Other outcome measures were recorded daily during neurocritical care unit stay (e.g., mechanical ventilation, extubation trials, adverse events), at hospital discharge, and by telephone interview with patients, relatives, or caregivers at 6 months.

Patients were selected for our post hoc analysis as follows:

1. ST group: All SETPOINT2 patients treated with surgical tracheostomy.
2. DT group: For each patient undergoing ST, a control set of patients undergoing DT with the same stroke subtype (AIS, ICH, SAH) and SETPOINT2 randomization status was identified, and the two closest matches based on age, premorbid functional status (mRS score), and baseline SETscore were selected after calculation of a propensity score via a logistic regression model.

The SETscore consists of three areas of assessment: (1) neurological function (dysphagia, observed aspiration, Glasgow Coma Scale < 10), (2) neurological lesion (brainstem, space-occupying cerebellar, ischemic infarct > 2/3 middle cerebral artery territory, ICH volume > 25 ml, diffuse lesion, hydrocephalus), and (3) general organ function/procedure (neurointervention/surgical intervention, additional respiratory disease, PaO2/FiO2 < 150, acute physiology score > 20, lung injury score > 1).

### Statistical Methods

Patient data for the two tracheostomy groups (ST group vs. DT group) were described using appropriate summary statistics for the respective empirical distributions (mean and standard deviation for continuous variables, median and interquartile range for scores, absolute and relative frequencies for categorical variables). Descriptive *p* values were derived from Mann–Whitney’s *U*-test for continuous variables and from *χ*^2^ tests for categorical variables.

Kaplan–Meier product-limit estimates were produced to compare the survival curves of the two tracheostomy groups over time, and a descriptive *p* value was derived via a log-rank test. The time to decannulation or death is presented in cumulative incidence plots for both tracheostomy groups. Finally, the association of tracheostomy type with the decannulation probability was investigated together with the covariates type of tracheostomy, SETPOINT2 randomization status, SETscore, stroke type (AIS, ICH, SAH), age, and premorbid functional status (mRS) by fitting a logistic regression model.

Statistical analyses were performed using the R software package (R Foundation for Statistical Computing, Austria. https://www.R-procject.org).

## Results

ST was performed in 41 of 307 tracheotomized SETPOINT2 patients. The comparison group of 82 patients with DT was derived from the SETPOINT2 trial cohort after propensity score matching for stroke type, randomization status, patient age, premorbid functional status (mRS), and SETscore.

Median patient age at stroke onset (59 years), premorbid functional status, and Glasgow Coma Scale score on hospital admission were comparable in both groups (Table 1). Included patients had AIS (17%), ICH (51%), and SAH (32%). Patients with AIS had a National Institutes of Health stroke scale score on admission of 19 (ST group IQR 13–35; DT group IQR 14–26), and stroke localization was predominantly supratentorial in both groups. In the subgroup of patients with ICH, the median ICH score was 3 (IQR 2–3), and 69% of patients had moderate to large ICH volumes (> 30 ml). In patients with SAH, clinical severity on admission according to the World Federation of Neurosurgical Societies scale was comparable in the two groups, however, using Fisher imaging criteria of SAH grading, patients with DT were more severely affected. The median SETscore, validated to estimate the need for prolonged mechanical ventilation, and the acute physiology score, indicating the severity of organ dysfunctions, were similar in the ST and the DT group.

**Table 1:** Baseline patient characteristics

Variable	ST group	DT group	Total		*p* value
	(*n* = 41)	(*n* = 82)	(*n* = 123)		
Patient age (years)					
Median (IQR)	58 (51–67)	60 (50–68)	59 (51–68)		0.974^a^
Sex					
Male	17 (41.5%)	40 (48.8%)	57 (46.3%)		0.443^b^
Female	24 (58.5%)	42 (51.2%)	66 (53.7%)		
Premorbid mRS					
0	31 (75.6%)	67 (81.7%)	98 (79.7%)		0.428^b^
≥ 1	10 (24.4%)	15 (18.3%)	25 (20.3%)		
GCS on admission					
Median (IQR)	6 (3–9)	6 (3–9)	6 (3–9)		0.806^a^
Stroke type					
AIS	7 (17.1%)	14 (17.1%)	21 (17.1%)		> 0.999^b^
ICH	21 (51.2%)	42 (51.2%)	63 (51.2%)		
SAH	13 (31.7%)	26 (31.7%)	39 (31.7%)		
AIS location					
Supratentorial					
Yes	7 (100.0%)	10 (71.4%)	17 (81.0%)		0.116^b^
No	0 (0.0%)	4 (28.6%)	4 (19.0%)		
Infratentorial					
Yes	1 (14.3%)	4 (28.6%)	5 (23.8%)		0.469^b^
No	6 (85.7%)	10 (71.4%)	16 (76.2%)		
NIHSS on admission					
*n*	7	13	20		0.874^a^
Median (IQR)	19 (13–35)	19 (14–26)	19 (14–26)		
ICH location					
Supratentorial					
Yes	14 (66.7%)	32 (76.2%)	46 (73.0%)		0.422^b^
No	7 (33.3%)	10 (23.8%)	17 (27.0%)		
Infratentorial					
Yes	7 (33.3%)	12 (28.6%)	19 (30.2%)		0.698^b^
No	14 (66.7%)	30 (71.4%)	44 (69.8%)		
ICH Volume					
≤ 30 cm^3^	7 (33.3%)	12 (29.3%)	19 (30.6%)		0.742^b^
> 30 cm^3^	14 (66.7%)	29 (70.7%)	43 (69.4%)		
ICH score (calculated)					
*n*	16	29	45		0.980^a^
Median (IQR)	3 (2–3)	3 (2–3)	3 (2–3)		
WFNS scoring					
Grade II	1 (7.7%)	1 (3.8%)	2 (5.1%)		0.829^a^
Grade III	2 (15.4%)	5 (19.2%)	7 (17.9%)		
Grade IV	3 (23.1%)	5 (19.2%)	8 (20.5%)		
Grade V	7 (53.8%)	15 (57.7%)	22 (56.4%)		
Fisher Scale scoring					
Grade I	0 (0.0%)	0 (0.0%)	0 (0.0%)		< 0.001^a^
Grade II	2 (15.4%)	1 (3.8%)	3 (7.7%)		
Grade III	7 (53.8%)	2 (7.7%)	9 (23.1%)		
Grade IV	4 (30.8%)	23 (88.5%)	27 (69.2%)		
SETscore					
Median (IQR)	15 (12–19)	15 (13–18)	15 (13–19)		0.790^a^
Acute physiology score					
*n*	33	59	92		0.350^a^
Missing	8 (19.5%)	23 (28.0%)	31 (25.2%)		
Median (IQR)	18 (15–23)	18 (13–20)	18 (14–22)		

*AIS* acute ischemic stroke, *DT* dilational tracheostomy, *GCS* Glasgow Coma Scale, *ICH* intracerebral hemorrhage, *IQR* interquartile range, *mRS*, modified Rankin Scale, *NIHSS*, National Institute of Health Stroke Scale, *SAH* subarachnoidal hemorrhage, *SETscore* stroke-related early tracheostomy score, *SD* standard deviation, *ST* surgical tracheostomy, *WFNS* World Federation of Neurosurgical Societies

^a^Mann-Whitney *U*-test

^b^Pearson’s *χ*^2^ test

In about 1 of 6 intubated patients in both groups, at least one extubation trial was performed before tracheostomy (Table 2). Early tracheostomy (≤ 5 days) was performed in 46% of the patients in ST and DT groups according to randomization in the SETPOINT2 trial.

**Table 2:** Treatment characteristics

Variable	ST group	DT group	Total	*p* value
	(*n* = 41)	(*n* = 82)	(*n* = 123)	
Extubation trial				
Yes	7 (17.1%)	15 (18.3%)	22 (17.9%)	0.868^a^
No	34 (82.9%)	67 (81.7%)	101 (82.1%)	
Randomization status				
Early tracheostomy	19 (46.3%)	38 (46.3%)	57 (46.3%)	> 0.999^a^
Standard therapy/late tracheostomy	22 (53.7%)	44 (53.7%)	66 (53.7%)	
Time from intubation to tracheostomy (days)				
*n*	41	82	123	0.025^b^
Mean (SD)	9.6 ± 5.6	7.5 ± 3.9	8.2 ± 4.6	
Median (IQR)	9 (5–12)	9 (4–11)	9 (4–11)	
Duration of sedation (days)				
*n*	33	59	92	0.750^b^
Missing (*n*)	8 (19.5%)	23 (28.0%)	31 (25.2%)	
Median (IQR)	9 (4–16)	8 (4–12)	8 (4–13.5)	
Days with mechanical ventilation				
*n*	17	41	58	0.008^b^
Missing (*n*)	24 (58.5%)	41 (50.0%)	65 (52.8%)	
mean (SD)	22.3 ± 10.0	15.8 ± 7.9	17.7 ± 9.0	
Median (IQR)	19 (17 – 24)	14 (11 – 19)	17 (12 – 22)	
Time from tracheostomy to end of ventilation (days)				
*n*	17	41	58	0.041^b^
Missing (*n*)	24 (58.5%)	41 (50.0%)	65 (52.8%)	
Mean (SD)	12.6 ± 9.8	8.1 ± 8.0	9.4 ± 8.7	
Median (IQR)	9 (6–17)	5 (2–12)	8 (2–14)	
Time from admission to ICU discharge (days)				
*n*	41	82	123	0.047^b^
Mean (SD)	23.3 ± 11.9	19.1 ± 8.3	20.5 ± 9.8	
Median (IQR)	23 (16–27)	17 (13–24)	18 (14–25)	
Time from admission to hospital discharge (days)				
*n*	41	82	123	0.246^b^
Mean (SD)	34.7 ± 30.3	26.5 ± 18.8	29.2 ± 23.5	
Median (IQR)	24 (18–37)	22 (16–32)	23 (16–33)	
Neurosurgical operation				
Yes	23 (69.7%)	38 (64.4%)	61 (66.3%)	0.607^a^
No	10 (30.3%)	21 (35.6%)	31 (33.7%)	
Missing	8	23	31	
Episodes with elevated ICP				
Yes	13 (32.5%)	14 (19.4%)	27 (24.1%)	0.122^a^
No	27 (67.5%)	58 (80.6%)	85 (75.9%)	
Missing	1	10	11	
Withdrawal of care (hospital)				
No withdrawal	37 (90.2%)	74 (90.2%)	111 (90.2%)	> 0.999^a^
Withdrawal	4 (9.8%)	8 (9.8%)	12 (9.8%)	
Discharge destination				
Home	1 (2.9%)	1 (1.4%)	2 (1.9%)	0.788^a^
Hospital	0 (0.0%)	0 (0.0%)	0 (0.0%)	
Rehab center	22 (62.9%)	47 (68.1%)	69 (66.3%)	
Long-term care facility	9 (25.7%)	13 (18.8%)	22 (21.2%)	
Other	3 (8.6%)	8 (11.6%)	11 (10.6%)	
Missing	6	13	19	
Mechanically ventilated at hospital discharge				
Yes	23 (65.7%)	43 (61.4%)	66 (62.9%)	0.668^a^
No	12 (34.3%)	27 (38.6%)	39 (37.1%)	
Missing	6	12	18	

*DT* dilational tracheostomy, *ICP* intracranial pressure, *ICU* intensive care unit, *IQR* interquartile range, *SD* standard deviation, *ST* surgical tracheostomy

^a^Pearson’s *χ*^2^ test

^b^Mann-Whitney’s *U*-test

Tracheostomy in patients undergoing ST was performed later compared with patients undergoing DT (median 9 [IQR 5–12] vs. 9 [IQR 4–11] days after intubation, *p* = 0.025).

Duration of sedation during intensive care unit (ICU) stay was not different in both groups, however, patients with ST were mechanically ventilated for longer than patients with DT (median 19 [IQR 17–24] vs.14 [IQR 11–19] days, *p* = 0.008). Accordingly, a longer ICU stay was observed after ST compared to DT (median 23 [IQR 16–27] vs. 17 [IQR 13–24] days, *p* = 0.047). However, the overall length of hospital stay was not significantly different in surgically and dilationally treated patients. These results were confirmed after adjusting for matching factors by multivariable linear regression (Supplemental Table 1).

During the hospital stay, early neurosurgical interventions were conducted in about two of three patients in each group. Episodes of elevated intracranial pressure (ICP) were observed in both groups (ST group 33% vs. DT group 19%, *p* = 0.122). Most patients were discharged to rehabilitation (ST group 63% vs. DT group 68%) and most patients in both groups were still mechanically ventilated at hospital discharge (ST group 66% vs. DT group 61%, *p* = 0.668). Mortality rate at ICU discharge was similar in the two groups (ST group 15% vs. DT group 12%, *p* = 0.705).

The primary outcome measure decannulation was observed in 56% of patients undergoing ST and in 61% of dilationally treated patients within 6 months after tracheostomy (*p* = 0.671; Table 3). However, in patients with available data on the decannulation date, the time to decannulation was significantly longer after ST compared with DT (81 [IQR 66–149] vs. 58 [IQR 32–77] days, *p* = 0.004). Of surviving patients with available cannulation status, 8 of 30 (27%) in the ST group and 10 of 55 (18%) in the DT group were cannulated at 6 months (*p* = 0.360).

**Table 3:** Decannulation, safety, and outcomes at hospital discharge and at 6 months

Variable	ST group	DT group	Total	*p* value
	(*n* = 41)	(*n* = 82)	(*n* = 123)	
Decannulation				
Yes	22 (56.4%)	46 (60.5%)	68 (59.1%)	0.671^a^
No	17 (43.6%)	30 (39.5%)	47 (40.9%)	
Missing	2	6	8	
Time to decannulation (days)				
*n*	22	40	62	0.004^b^
Missing	0	6	6	
Mean (SD)	110.4 ± 67.6	69 ± 62.6	83.7 ± 66.9	
Median (IQR)	81 (66 – 149)	58 (32–77)	66 (42–110)	
Death during hospital stay				
Alive	35 (85.4%)	72 (87.8%)	107 (87.0%)	0.705^a^
Dead	6 (14.6%)	10 (12.2%)	16 (13.0%)	
mRS score at 6 months				
*n*	39	79	118	0.514^b^
Missing	2 (4.9%)	3 (3.7%)	5 (4.1%)	
Median (IQR)	5 (4–6)	5 (3–6)	5 (4–6)	
mRS score at 6 months, dichotomized 0–3 vs. 4–6				
*n*	39	79	115	0.240^a^
0–3	7 (17.9%)	22 (27.8%)	29 (24.6%)	
4–6	32 (82.1%)	57 (72.2%)	89 (75.4%)	
mRS score at 6 months, dichotomized 0–4 vs. 5–6				
*n*	39	79	118	0.468^a^
0–4	16 (41.0%)	38 (48.1%)	54 (45.8%)	
5–6	23 (59.0%)	41 (51.9%)	64 (54.2%)	
Death at 6 months				
Alive	30 (75.0%)	56 (70.9%)	86 (72.3%)	0.636^a^
Dead	10 (25.0%)	23 (29.1%)	33 (27.7%)	
Missing	1	3	4	
Withdrawal of care (6 months)				
No withdrawal	34 (87.2%)	65 (82.3%)	99 (83.9%)	0.469^a^
Withdrawal	5 (12.8%)	14 (17.7%)	19 (16.1%)	
Missing	2	3	5	
Destination at 6 months				
Home	11 (34.4%)	28 (44.4%)	39 (41.1%)	0.570^a^
Hospital	2 (6.2%)	2 (3.2%)	4 (4.2%)	
Rehab center	11 (34.4%)	13 (20.6%)	24 (25.3%)	
Long-term care	5 (15.6%)	13 (20.6%)	18 (18.9%)	
Other	3 (9.4%)	7 (11.1%)	10 (10.5%)	
Required a caregiver at 6 months				
Yes	25 (67.6%)	35 (53.8%)	60 (58.8%)	0.312^a^
No	0 (0.0%)	4 (6.2%)	4 (3.99%)	
Dead	10 (27.0%)	23 (35.4%)	33 (32.4%)	
Lost to follow-up	2 (5.4%)	3 (4.6%)	5 (4.9%)	
Missing	4	17	21	

*DT* dilational tracheostomy, *IQR* interquartile range, *mRS* modified Rankin Scale, *SD* standard deviation, *ST* surgical tracucheostomy

^a^Pearson’s *χ*^2^ test

^b^Mann-Whitney’s *U*-test

**Fig. 1:**
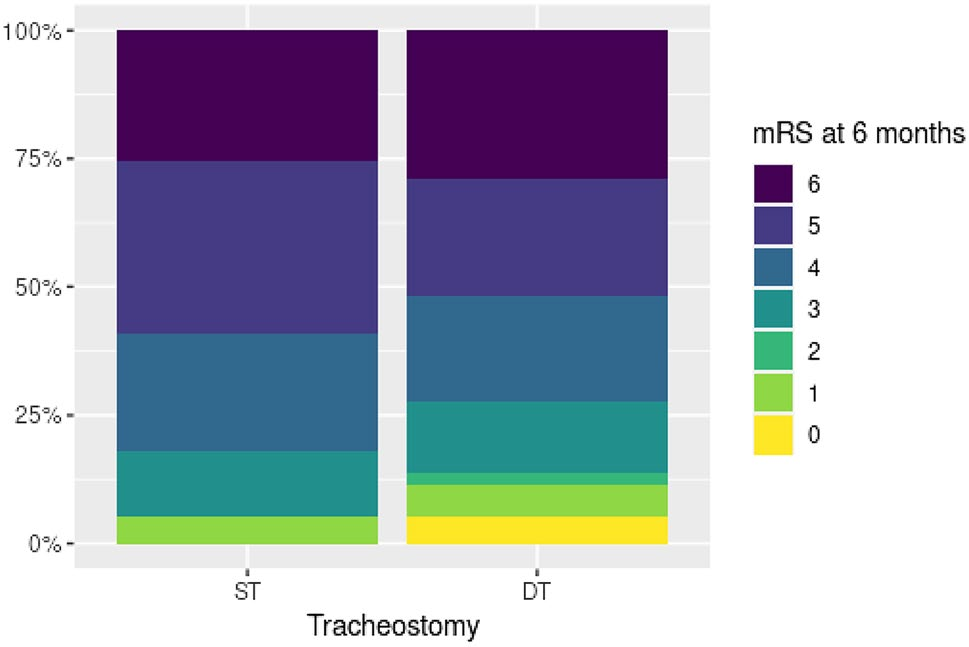
Modified Rankin Scale scores at 6 months. Figure 1 shows the functional outcome of surgically and dilationally tracheotomized patients at 6 months according to the modified Rankin Scale (scores 0–6, ranging from 0 [no symptoms] to 5 [severe disability]; 6 = death). Functional outcome and mortality at 6 months were comparable in both patient groups. *DT* dilational tracheostomy, *mRS* modified Rankin Scale, *ST* surgical tracheostomy

**Fig. 2:**
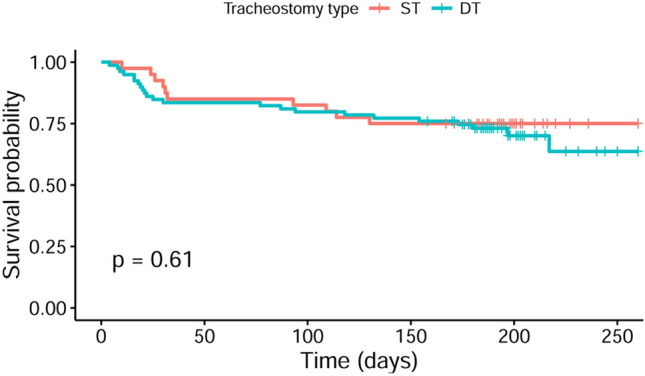
Time to event-analysis for probability of survival at 6 months. In Fig. 2, the Kaplan–Meier curves were shown for survival of patients with surgical tracheostomy (ST) and patients with dilational tracheostomy (DT), revealing comparable survival probabilities for patients of both treatment groups at 6 months

Functional outcome at 6 months after stroke onset according to the mRS was not significantly different in both goups (*p* = 0.514) and most surviving patients were moderately to severely disabled (Fig. 1). The proportions of surviving patients without very severe disability, translating to a mRS score of 0–4, were not significantly different between the two groups. The proportions of patients living at home at 6 months and the quality of life of surviving patients, according to EuroQol 5 dimensions rating, were comparable. Caregivers of both patient groups rated their burden due to patient care comparably high. Likewise, mortality rates at 6 months were not different in patients with ST and patients with DT (*p* = 0.636; Fig. 2).

The observed rate of adverse events and severe adverse events was low in the two groups, however, intrahospital infection rates attributable to tracheostomy were observed more frequently in surgically compared to dilationally treated patients (14.6% vs. 1.2%, *p* = 0.002; Table 3, Supplemental Table 2).

**Fig. 3:**
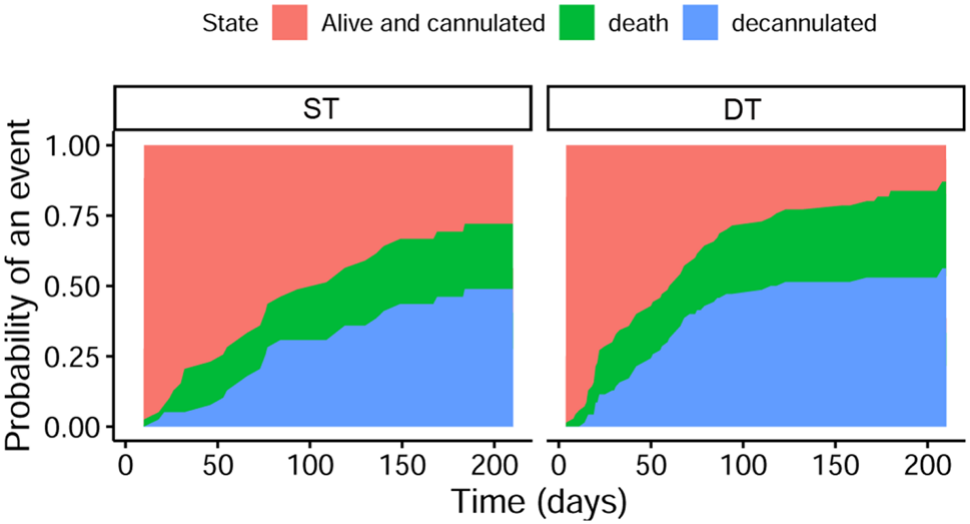
Cumulative incidence functions for probability of decannulation or death at 6 months. This figure illustratesprobabilities for decannulation, taking the probable competing event death into account. Figure 3 shows that decannulation rates are comparable in patients treated with surgical tracheostomy (ST) and patients treated with dilational tracheostomy (DT), but patients with DT were decannulated earlier and had a significantly higher probability of cannulation-free time within the first months after tracheostomy

The results of the cumulative incidence functions, including the competing events death and decannulation, are shown in Fig. 3. This figure illustrates that patients with ST have a significantly lower probability for canulation-free time compared with dilationally treated patients within the first 6 months after tracheostomy.

The multivariable regression model, based on data of both ST and DT groups, revealed that the type of tracheostomy (surgical vs. dilational) was not associated with the probability of decannulation within 6 months (Supplemental Table 3). A higher baseline SETscore was identified as negative predictor of decannulation probability. Stroke type (AIS, ICH, and SAH), patient age, and premorbid functional status (mRS) were not significantly associated with decannulation in our model.

## Discussion

Our analysis of 123 ventilated tracheostomized patients with severe stroke revealed that decannulation rates and clinical outcomes at 6 months after stroke onset were not different if patients were tracheostomized surgically or dilationally. However, significant differences between the two treatment groups were observed for clinically important treatment and outcome aspects, including significantly longer time to tracheostomy, length of ICU stay, and time to decannulation in patients undergoing ST compared with patients undergoing DT.

Stroke-specific patient characteristics were similar in the comparison groups, which is reflected by a median SETscore of 15 and comparable clinical scores for stroke subtypes in both groups. Furthermore, patient characteristics and stroke severity was comparable to other cohorts of tracheotomized patients with stroke [8, 10].

The clinical outcome results of our analysis suggest that the choice of the tracheostomy method (surgical, dilational) has no important influence on functional status and survival 6 months after severe stroke. Furthermore, the rate of decannulation 6 months after stroke onset, which is thought to be associated, for example, with functional aspects (speech, swallowing, mobility) or the level of care, was not different in the two tracheostomy groups. In earlier reports, decannulation rates for surviving patients with acute brain injury including stroke at 6–12 months varied between for 49% and 95% (all tracheotomized patients) and 34% and 82% (surviving patients), respectively [9]. Accordingly, the observed decannulation rates in the two analyzed groups at 6 months (56% and 61%, respectively) are in line with previous results. Sufficient data to specifically compare decannulation rates after ST and DT in patients with stroke are not available in the literature. Furthermore, the mortality rate at 6 months of 28% in the analyzed SETPOINT2 cohort was comparable to studies reporting mortality rates between 30 and 46% for tracheotomized patients with stroke [8, 10, 12]. In a Cochrane review comparing surgically and dilationally treated ICU patients, early tracheostomy-related mortality was low (6/257 patients) and did not differ between patients with ST and patients with DT [3]. Mortality rate is important in this context, as death may prevent decannulation as a competing event.

Especially from the perspective of patients and caregivers, the cannulation-free time might serve as a surrogate compound parameter, for example, for functional outcome, quality of life, and need for care. Time to decannulation was significantly shorter in the DT group (median 58 days) compared with surgically treated patients (median 81 days), which translated into more cannulation-free time and hence the potential to be associated with a better quality of life and need of lower level of care. However, quality of life was not rated differently in the ST and DT groups in our analysis. Of note, previous reports, including mixed groups of patients with stroke treated with ST or DT, revealed a range for time to decannulation of 72–89 days [8, 16]. Strategies of weaning of cannulation in the different settings (hospital, rehabilitation, outpatient) were not evaluated in our cohort, but weaning procedures may have a profound impact on decannulation frequencies. However, the observed overall shorter time to decannulation in dilationally treated patients is clinically relevant.

Importantly, our analysis revealed differences for intrahospital treatment parameters between the two groups related to mechanical ventilation. First, the observed longer time to tracheostomy; second, the longer duration of mechanical ventilation during hospital stay; third, the longer ICU stay in surgically tracheotomized patients. Hospital mortality rates were comparable between the two groups (ST group 15%, DT group 12%) and with previous reports on patients with tracheotomized stroke (0–28%) [9]. The longer time to ST, which may, in part, explain the longer mechanical ventilation and the longer ICU stay, is not explained by baseline patient and treatment characteristics, for example, extubation trials or neurosurgical interventions before tracheostomy. Availability of trained surgical personal or operating room capacities might have led to a delay of ST.

Adverse events associated with tracheostomy were also rarely and similarly often observed in both SETPOINT2 groups, however, early infections were found significantly more often in the patients with ST. In line with our results, tracheal stoma wound infections were observed more frequently among patients undergoing ST (84/473) compared with patients undergoing DT (18/463), RR 0.24 [95% confidence interval 0.15–0.37] in other cohorts [3]. In-hospital bleeding complications were observed in 4.9% (ST group) and in 3.7% (DT group) (*p* = 0.747). In comparison, major bleeding episodes were reported in 8% of patients with ST (39/488) and 6% of patients with DT (29/496) for other cohorts according to a previous meta-analysis [3]. Early life-threatening adverse events (< 24 h after tracheostomy) related to tracheostomy were rare and not different between patients with ST and patients with DT.

During ICU treatment, episodes of elevated ICP were observed not significantly more often in surgically tracheostomized patients of our cohort. Numerical differences in ICP episodes (ST group 33% vs. DT group 19%) did not clinically translate into a higher mortality rate of patients with ST at hospital discharge or at 6 months.

At follow-up after 6 months, specific, late tracheostomy-related complications were reported very rarely for our SETPOINT2 groups, for example, one tracheal stenosis, one fistula, and no scar problems were reported. Earlier data suggest that these late complications, which might have an considerable impact on quality of life, were more frequently observed in other tracheostomy cohorts [17, 18]. It is of importance, that in our cohort information on late tracheostomy complications were mainly not available for deceased patients.

In the two comparison groups, information on quality of life of about 2/3 of patients in each group were available, and patients rated their quality of life similarly in both groups. Furthermore, caregivers of both groups rated their burden of patient care similarly. This is partially surprising, as surviving patients of the ST group had significantly less cannula-free time during the first 6 months after tracheostomy and were more often persistantly cannulated at 6 months. However, as functional outcome according to mRS score was not significantly different in the two groups, overall disability might have overwhelmed other considerations in the evaluations of patients and caregivers.

In the combined cohort of surgically and dilatonally treated patients with stroke, a higher SETscore was identified as negative predictor for decannulation. Patient age was barely not significant as a predictor in our cohort, however, age was identified as a negative predictor in previous studies including ventilated patients with stroke [8, 9, 16]. Type of stroke, premorbid functional status (mRS) and type of tracheostomy were also not predictive for decannulation in our cohort.

Several limitations of our analysis need to be considered. SETPOINT2 was an investigator-initiated trial with limited funding, allowing infrequent site monitoring, a pragmatic data sampling via simple electronic case report form and outcome assessment via telephone interview, including the primary outcome of decannulation. This may explain the partially missing data for some of the outcome measures and the relatively low number of reported tracheostomy-related adverse events. Outcome evaluation in patients with stroke needs to include several dimensions, preferentially including the perspectives of patients, relatives, and caregivers. Retrieving the feedback of patients affected by a severe stroke is often limited by their residual neurological deficits, profound communication barriers, and logistical issues, especially if patients are cared for outside their former homes. These limitations may lead to a reporting bias, favoring patients without relevant reporting barriers. In this context, the limitations of a retrospective approach of our analysis needs to be considered.

The sample size of surgically tracheotomized SETPOINT2 patients was rather small, however, together with the comparison group of patients with DT, this is one of the largest cohorts of tracheostomized patients with stroke with prospective outcome evaluation. As such, it adds to our understanding of the outcomes of patients undergoing tracheostomy after severe stroke.

As discussed previously, several aspects of the SETPOINT2 study design and conduct may have had a strong impact the results of our analysis. We included the SETPOINT2 randomization status in our matching approach to avoid a possible influence of randomization imbalances especially on intrahospital outcome measures including time to tracheostomy and length of hospital stay. As participating centers were selected by performing preferentially DT as standard of care, ST might have been a rescue strategy in several patients. However, the reasons for selecting the surgical method (relative contraindications to DT, rescue strategy after a trial of DT, availability of tracheostomy resources) were not prospectively recorded and not available for our analysis. Rescue ST could have been performed in patients with DT contraindications or less experienced personal. This may also explain higher early infections rates.

Furthermore, generalizability of our results is limited since SETPOINT2 was conducted in specialized Neuro-ICUs of mainly academic centers. Therefore, treatment strategies and clinical results for patients treated outside this setting may differ. A major limitation is that posthospital treatment, rehabilitational strategies and intensity of rehabilitation, and other post-rehabilitational care was not evaluated in detail.

We report one of the largest cohorts of tracheostomized patients with stroke with prospectively collected data on ST and DT, treated within a multicenter clinical trial and at specialized neurological/neurosurgical ICUs. Furthermore, a structured outcome assessment including blinded mRS-rating at 6 months was performed. The primary outcome measure (decannulation) and the main competing measure (death) are robust clinical parameters.

## Conclusions

In conclusion, in this post hoc analysis of a randomized trial, ST and DT in mechanically ventilated patients with stroke are associated with comparable rates of decannulation, mortality, and functional outcome at 6 months in selected SETPOINT2 patients with severe ischemic and hemorrhagic stroke. Quality of life and burden of care were rated equally by patients and their caregivers in both treatment groups. However, the results for several clinically important measures appear to support dilational over surgical trachestomy in patients with severe stroke. Patients with DT had lower early infection rates and were decannulated earlier. Further studies should evaluate decannulation strategies, long-term adverse events of tracheostomy, and patient-centered as well as caregiver-centered outcome measures in more detail.

### Supplementary Information

Below is the link to the electronic supplementary material.

Supplementary file1 (DOCX 28 kb)
