# Supplementary material for: Surgical Versus Dilational Tracheostomy in Patients with Severe Stroke: A SETPOINT2 Post hoc Analysis
Source: Neurocrit Care. 2024 Jan 30;41(1):146–55. doi: 10.1007/s12028-023-01933-9 (PMC11335838; doi:10.1007/s12028-023-01933-9)
Supplement: Supplementary file 1 — Supplementary file1 (DOCX 28 kb) [file 12028_2023_1933_MOESM1_ESM.docx]

**Supplement**

**Title:** **Surgical vs. Dilational Tracheostomy in Patients with Severe Stroke - a SETPOINT2 *post hoc* - Analysis**

**Authors:** Hauke Schneider MD, Jan Meis, Christina Klose, Peter Ratzka MD, Wolf-Dirk Niesen MD, David B. Seder MD, Julian Bösel MD; for the SETPOINT2 and IGNITE study groups

**Supplemental tables**

**Supplemental Table 1:** Multivariable linear models for intrahospital treatment and outcome measures adjusted for the propensity score matching factors

###

### Time from intubation to tracheostomy (days)

| Variable | Coefficient | 2.5% CI | 97.5% CI | p-value |
| --- | --- | --- | --- | --- |
| (Intercept) | 6.5636 | 2.3034 | 10.8239 | 0.003 |
| **DT** | **-2.1376** | **-3.3517** | **-0.9234** | **<0.001** |
| Randomization group late trachesotomy | 6.6286 | 5.4626 | 7.7945 | <0.001 |
| SETScore | -0.0211 | -0.1699 | 0.1277 | 0.780 |
| ICH | 0.3899 | -1.2625 | 2.0422 | 0.641 |
| SAH | 1.3824 | -0.3988 | 3.1636 | 0.127 |
| Patient age | -0.0152 | -0.0666 | 0.0362 | 0.559 |
| Premorbid mRS | 0.3630 | -1.2061 | 1.9322 | 0.648 |

### Duration of sedation (days)

| Variable | Coefficient | 2.5% CI | 97.5% CI | p-value |
| --- | --- | --- | --- | --- |
| (Intercept) | 12.5725 | 1.4709 | 23.6740 | 0.027 |
| **DT** | **-0.6096** | **-3.6356** | **2.4164** | **0.690** |
| Randomization group late trachesotomy | 2.1044 | -0.9058 | 5.1145 | 0.168 |
| SETScore | -0.0127 | -0.3822 | 0.3567 | 0.945 |
| ICH | -1.6697 | -6.0770 | 2.7377 | 0.453 |
| SAH | 2.8534 | -1.6714 | 7.3782 | 0.213 |
| Patient age | -0.0650 | -0.1970 | 0.0670 | 0.330 |
| Premorbid mRS | 2.9104 | -1.6942 | 7.5151 | 0.212 |

### Days with mechanical ventilation

| Variable | Coefficient | 2.5% CI | 97.5% CI | p-value |
| --- | --- | --- | --- | --- |
| (Intercept) | 14.5013 | -1.8772 | 30.8797 | 0.081 |
| **DT** | **-5.7383** | **-10.7732** | **-0.7034** | **0.026** |
| Randomization group late trachesotomy | 2.4004 | -2.0467 | 6.8475 | 0.283 |
| SETScore | 0.5011 | -0.0852 | 1.0874 | 0.092 |
| ICH | 3.0056 | -4.9995 | 11.0106 | 0.454 |
| SAH | 5.5243 | -2.6530 | 13.7017 | 0.181 |
| Patient age | -0.1167 | -0.3081 | 0.0746 | 0.226 |
| Premorbid mRS | 6.8240 | 1.4259 | 12.2221 | 0.014 |

### Time from tracheostomy to end of ventilation (days)

| Variable | Coefficient | 2.5% CI | 97.5% CI | p-value |
| --- | --- | --- | --- | --- |
| (Intercept) | 6.2766 | -9.5891 | 22.1423 | 0.431 |
| **DT** | **-2.3815** | **-7.2587** | **2.4958** | **0.331** |
| Randomization group late trachesotomy | -4.4322 | -8.7401 | -0.1243 | 0.044 |
| SETScore | 0.4751 | -0.0928 | 1.0430 | 0.099 |
| ICH | 4.8430 | -2.9114 | 12.5975 | 0.216 |
| SAH | 5.3524 | -2.5689 | 13.2738 | 0.181 |
| Patient age | -0.1093 | -0.2947 | 0.0760 | 0.242 |
| Premorbid mRS | 6.4852 | 1.2561 | 11.7143 | 0.016 |

### Time from admission to ICU discharge (days)

| Variable | Coefficient | 2.5% CI | 97.5% CI | p-value |
| --- | --- | --- | --- | --- |
| (Intercept) | 15.3949 | 3.3700 | 27.4198 | 0.013 |
| **DT** | **-3.9863** | **-7.4134** | **-0.5591** | **0.023** |
| Randomization group late trachesotomy | 3.9694 | 0.6785 | 7.2604 | 0.019 |
| SETScore | 0.3609 | -0.0592 | 0.7809 | 0.092 |
| ICH | 4.9870 | 0.3231 | 9.6510 | 0.036 |
| SAH | 9.2630 | 4.2354 | 14.2906 | <0.001 |
| Patient age | -0.1136 | -0.2588 | 0.0316 | 0.124 |
| Premorbid mRS | 5.3209 | 0.8919 | 9.7500 | 0.019 |

### Time from admission to hospital discharge (days)

| Variable | Coefficient | 2.5% CI | 97.5% CI | p-value |
| --- | --- | --- | --- | --- |
| (Intercept) | 36.7640 | 6.1625 | 67.3655 | 0.019 |
| **DT** | **-8.1815** | **-16.9031** | **0.5401** | **0.066** |
| Randomization group late trachesotomy | 6.6675 | -1.7075 | 15.0424 | 0.118 |
| SETScore | 0.2449 | -0.8242 | 1.3139 | 0.651 |
| ICH | 9.4661 | -2.4029 | 21.3350 | 0.117 |
| SAH | 12.0135 | -0.7809 | 24.8080 | 0.065 |
| Patient age | -0.3160 | -0.6855 | 0.0534 | 0.093 |
| Premorbid mRS | 2.0644 | -9.2068 | 13.3356 | 0.717 |

DT, dilational tracheostomy; ICH, intracerebral hemorrhage; SAH, subarachnoid hemorrhage; mRS, modified Rankin Scale score; ICU, intensive care unit

**Supplemental Table 2:** Adverse events and severe adverse events

| Variable | ST group | DT group | Total | p |
| --- | --- | --- | --- | --- |
|  | (N=41) | (N=82) | (N=123) |  |
| **Periprocedural adverse events (< 2 hours) - Ventilation** |  |  |  |  |
| Yes | 2 (4.9%) | 2 (2.4%) | 4 (3.3%) | 0.472^chi2^ |
| No | 39 (95.1%) | 80 (97.6%) | 119 (96.7%) |  |
| **Periprocedural adverse events - Bleeding** |  |  |  |  |
| Yes | 2 (4.9%) | 3 (3.7%) | 5 (4.1%) | 0.747^chi2^ |
| No | 39 (95.1%) | 79 (96.3%) | 118 (95.9%) |  |
| **Periprocedural adverse events - Local trauma** |  |  |  |  |
| Yes | 0 (0.0%) | 0 (0.0%) | 0 (0.0%) |  |
| No | 41 (100.0%) | 82 (100.0%) | 123 (100.0%) |  |
| **Periprocedural adverse events – Cerebral compromise** |  |  |  |  |
| Yes | 1 (2.4%) | 0 (0.0%) | 1 (0.8%) | 0.156^chi2^ |
| No | 40 (97.6%) | 82 (100.0%) | 122 (99.2%) |  |
| **Early tracheostomy-related adverse events (from 2 h to ICU discharge) – all** |  |  |  |  |
| Yes | 6 (14.6%) | 1 (7.3%) | 7 (9.8%) | 0.002^chi2^ |
| No | 35 (85.4%) | 81 (92.7%) | 116 (90.2%) |  |
| **Early tracheostomy-related adverse events - infections** |  |  |  |  |
| Yes | 6 (14.6%) | 1 (1.2%) | 7 (5.7%) | 0.002^chi2^ |
| No | 35 (85.4%) | 81 (98.8%) | 116 (94.3%) |  |
| **Early tracheostomy-related adverse events – tracheostomy tube** |  |  |  |  |
| Yes | 2 (4.9%) | 0 (0.0%) | 2 (1.6%) | 0.044^chi2^ |
| No | 39 (95.1%) | 82 (100.0%) | 121 (98.4%) |  |
| **Late tracheostomy-related adverse events (from hospital discharge to follow-up) - all** |  |  |  |  |
| Yes | 2 (5.9%) | 5 (8.2%) | 7 (7.4%) | 0.679^chi2^ |
| No | 32 (94.1%) | 56 (91.8%) | 88 (92.6%) |  |
| (Missing) | 7 | 21 | 28 |  |
| **Late tracheostomy-related adverse events – infection at tracheostomy site** |  |  |  |  |
| Yes | 0 (0.0%) | 0 (0.0%) | 2 (0.0%) |  |
| No | 33 (100.0%) | 61 (100.0%) | 94 (100.0%) |  |
| (Missing) | 8 | 21 | 29 |  |
| **Late tracheostomy-related adverse events – scaring / disturbed wound healing at tracheostomy site** |  |  |  |  |
| Yes | 0 (0.0%) | 3 (4.9%) | 3 (3.2%) | 0.195^chi2^ |
| No | 33 (100.0%) | 58 (95.1%) | 91 (96.8%) |  |
| (Missing) | 8 | 21 | 29 |  |
| **Late tracheostomy-related adverse events – Tracheocutanous fistula** |  |  |  |  |
| Yes | 0 (0.0%) | 1 (1.6%) | 1 (1.1%) | 0.453^chi2^ |
| No | 34 (100.0%) | 60 (98.4%) | 94 (98.9%) |  |
| (Missing) | 7 | 21 | 28 |  |
| **Late tracheostomy-related adverse events – tracheal instability** |  |  |  |  |
| Yes | 0 (0.0%) | 0 (0.0%) | 0 (0.0%) |  |
| No | 34 (100.0%) | 61 (100.0%) | 95 (100.0%) |  |
| (Missing) | 7 | 21 | 28 |  |
| **Late tracheostomy-related adverse events – clinically relevant tracheal stenosis** |  |  |  |  |
| Yes | 1 (2.9%) | 0 (0.0%) | 1 (1.1%) | 0.178^chi2^ |
| No | 33 (97.1%) | 61 (100.0%) | 94 (98.9%) |  |
| (Missing) | 7 | 21 | 28 |  |
| **Late tracheostomy-related adverse events – Complicated change of cannula** |  |  |  |  |
| Yes | 0 (0.0%) | 1 (1.6%) | 1 (1.1%) | 0.453^chi2^ |
| No | 34 (100.0%) | 60 (98.4%) | 94 (98.9%) |  |
| (Missing) | 7 | 21 | 28 |  |
| **Late tracheostomy-related adverse events – need vor surgical revision of stoma** |  |  |  |  |
| Yes | 1 (3.0%) | 0 (0.0%) | 1 (1.1%) | 0.172^chi2^ |
| No | 32 (97.0%) | 61 (100.0%) | 93 (98.9%) |  |
| (Missing) | 8 | 21 | 29 |  |
| ^chi2^Pearson's chi-squared test | | | | |
|  | | | | |
| ^MWU^Mann-Whitney's U-test | | | | |
|  | | | | |

ST, surgical tracheostomy; DT, dilational tracheostomy

**Supplemental Table 3**: **Multivariable logistic regression model for prediction of decannulation status at 6 months**

| **Variable** | **Odds ratio** | **2.5% CI** | **97.5% CI** | **p-value** |
| --- | --- | --- | --- | --- |
| (Intercept) | 57.9907 | 2.6888 | 1583.0958 | 0.012 |
| DT (compared to ST) | 1.2331 | 0.5278 | 2.8757 | 0.626 |
| Randomization to standard therapy / late tracheostomy | 2.4265 | 1.0838 | 5.5824 | 0.033 |
| SETScore | 0.8903 | 0.7995 | 0.9871 | 0.029 |
| ICH (compared to AIS) | 0.4977 | 0.1511 | 1.5349 | 0.233 |
| SAH (compared to AIS) | 1.1538 | 0.3262 | 3.9934 | 0.821 |
| Patient age | 0.9661 | 0.9301 | 1.0016 | 0.066 |
| Premorbid mRS | 0.8285 | 0.2852 | 2.4039 | 0.727 |

ST, surgical tracheostomy; DT, dilational tracheostomy; ICH, intracerebral hemorrhage; SAH, subarachnoid hemorrhage; AIS, acute ischemic stroke; mRS, modified Rankin Scale score; ICU, intensive care unit

**Supplemental Table 4: Tracheostomy type performed by participating SETPOINT2 centers (all tracheostomies)**

| **Center** | **Surgical tracheostomy**  **(n=41)** | **Dilational tracheostomy (n=266)** |
| --- | --- | --- |
| Heidelberg Neurology | 9 (12%) | 63 (88%) |
| Freiburg | 0 (0%) | 10 (100%) |
| Portland | 0 (0%) | 28 (100%) |
| Kassel | 3 (18%) | 14 (82%) |
| Köln | 0 (0%) | 2 (100%) |
| Hamburg | 0 (0%) | 1 (100%) |
| Houston | 0 (0%) | 21 (100%) |
| New York, Columbia | 1 (50%) | 1 (50%) |
| Berlin | 2 (6%) | 29 (94%) |
| Leipzig | 0 (0%) | 0 (0%) |
| Regensburg | 0 (0%) | 0 (0%) |
| Heidelberg Neurosurgery | 4 (27%) | 11 (73%) |
| Dresden | 0 (0%) | 1 (100%) |
| Columbus Ohio | 0 (0%) | 17 (100%) |
| Fullerton California | 0 (0%) | 0 (0%) |
| Berlin, Charité BF | 1 (25%) | 3 (75%) |
| New Haven | 2 (22%) | 7 (78%) |
| Mainz | 0 (0%) | 0 (0%) |
| New York, Mt. Sinai | 0 (0%) | 2 (100%) |
| Jacksonville | 2 (33%) | 4 (67%) |
| Detroit | 0 (0%) | 2 (100%) |
| Baltimore | 9 (31%) | 20 (69%) |
| Durham NC | 1 (17%) | 5 (83%) |
| Philadelphia PA | 0 (0%) | 0 (0%) |
| Illinois | 2 (50%) | 2 (50%) |
| Richland WA | 0 (0%) | 8 (100%) |
| Honolulu | 0 (0%) | 0 (0%) |
| Cincinnati | 0 (0%) | 0 (0%) |
| Augsburg | 1 (12%) | 7 (88%) |
| Baltimore | 0 (0%) | 0 (0%) |
| Ann Arbor | 0 (0%) | 6 (100%) |
| Burlington | 0 (0%) | 0 (0%) |
| Houston BCM | 4 (80%) | 1 (20%) |
| Knoxville | 0 (0%) | 1 (100%) |
